# Prevalence of amino acid mutations in hepatitis C virus core and NS5B regions among Venezuelan viral isolates and comparison with worldwide isolates

**DOI:** 10.1186/1743-422X-9-214

**Published:** 2012-09-21

**Authors:** Rossana C Jaspe, Yoneira F Sulbarán, Maria Z Sulbarán, Carmen L Loureiro, Hector R Rangel, Flor H Pujol

**Affiliations:** 1 Laboratorio de Virología Molecular, Centro de Microbiología y Biología Celular, Instituto Venezolano de Investigaciones Científicas, Apdo 20632, Caracas 1020-A, Venezuela

**Keywords:** HCV, Core, NS5B, Mutation

## Abstract

**Background:**

Recent reports show that R70Q and L/C91M amino acid substitutions in the core from different hepatitis C virus (HCV) genotypes have been associated with variable responses to interferon (IFN) and ribavirin (RBV) therapy, as well to an increase of hepatocellular carcinoma (HCC) risk, liver steatosis and insulin resistance (IR). Mutations in NS5B have also been associated to IFN, RBV, nucleoside and non-nucleoside inhibitors drug resistance. The prevalence of these mutations was studied in HCV RNA samples from chronically HCV-infected drug-naïve patients.

**Methods:**

After amplification of core and NS5B region by nested-PCR, 12 substitutions were analyzed in 266 Venezuelan HCV isolates subtype 1a, 1b, 2a, 2c, 2b, 2j (a subtype frequently found in Venezuela) and 3a (n = 127 and n = 228 for core and NS5B respectively), and compared to isolates from other countries (n = 355 and n = 646 for core and NS5B respectively).

**Results:**

R70Q and L/C91M core substitutions were present exclusively in HCV G1b. Both substitutions were more frequent in American isolates compared to Asian ones (69% versus 26%, p < 0.001 and 75% versus 45%, p < 0.001 respectively). In Venezuelan isolates NS5B D310N substitution was detected mainly in G3a (100%) and G1a (13%), this later with a significantly higher prevalence than in Brazilian isolates (p = 0.03). The NS5B mutations related to IFN/RBV treatment D244N was mainly found in G3a, and Q309R was present in all genotypes, except G2. Resistance to new NS5B inhibitors (C316N) was only detected in 18% of G1b, with a significantly lower prevalence than in Asian isolates, where this polymorphism was surprisingly frequent (p < 0.001).

**Conclusions:**

Genotypical, geographical and regional differences were found in the prevalence of substitutions in HCV core and NS5B proteins. The substitutions found in the Venezuelan G2j type were similar to that found in G2a and G2c isolates. Our results suggest a high prevalence of the R70Q and L/C91M mutations of core protein for G1b and D310N substitution of NS5B protein for the G3a. C316N polymorphism related with resistance to new NS5B inhibitors was only found in G1b. Some of these mutations could be associated with a worse prognosis of the disease in HCV infected patients.

## Background

Around 170 million persons (3% of the world population) are infected with the hepatitis C virus (HCV). More than 350,000 people die from HCV-related liver diseases, including hepatocellular carcinoma (HCC), each year [1]. In Latin America around 7 million persons are infected with HCV and overall prevalence of HCV antibodies in non-Amerindian populations from South America is around 1% [2-4]. About 130 million people in the world are chronically infected with HCV and at risk of developing liver inflammation, fibrosis, cirrhosis and steatosis leading to HCC [1]. Furthermore, in these chronic patients HCV infection has an increased risk of type-2 diabetes mellitus or insulin resistance (IR) [5].

Seven HCV genotypes, and a large number of subtypes in each genotype, have been described. Genotypes 1, 2, and 3 have a worldwide distribution, and their prevalence varies according to the region of the world. In Venezuela, genotype 1 is the most frequently detected, followed by genotypes 2 and 3, however a significant reduction of HCV G1b circulation was observed in the last decade, with the increase in circulation of genotype G2j, a subtype quite rare in other countries, including the Americas [6,7].

While a vaccine for HCV is not available, many new antiviral agents are being tested to treat HCV infection, such as nucleoside (NIs) and non-nucleoside (NNIs) polymerase and protease inhibitors. The combination of pegylated interferon alpha (IFN) and ribavirin (RBV), plus new HCV NS3/4A serine protease inhibitors (boceprevir or telaprevir) is the current treatment for patients chronically infected with HCV [8]. HCV genotype is also a predictive parameter for sustained virological response (SVR). Infections with HCV genotype 1 are associated with the lowest therapeutic success [9].

Within the HCV genome, core is thought to be the most conserved protein; results of nucleotide and deduced amino acid sequence analysis across diverse strains of HCV reveal 81–88% nucleotide and 96% amino acid sequence homology [10,11]. The core gene region plays several essential roles in the HCV life cycle. Recent studies have revealed that amino acid substitutions in the core region of different HCV genotypes are associated with IR [12], increased HCC risk [13-17], variable responses to double (IFN/RBV) [18-24] and triple (telaprevir plus INF/RBV) therapy [25,26], and liver steatosis [27-30]. On the other hand, the region of the viral genome that codes for NS5B has been described as an important target in therapy with IFN/RBV, and nucleoside and non-nucleoside drugs [31]. Many drug-resistance mutations induced by antiviral treatment are located in this region of the genome [32-37].

In the present study, we analyzed core and NS5B polymorphisms/mutations which have been associated with non-responsiveness or a better response to IFN/RBV combination therapy, and/or resistance to polymerase inhibitor drugs, HCC, liver steatosis and IR, in chronically infected drug-naïve patients from Venezuela, and these polymorphisms/mutations were compared with worldwide HCV isolates.

## Results

Some relevant core and NS5B substitutions associated with treatment response, as well as, with a worse prognosis of the disease were analyzed. Four core substitutions [12-30] and eight NS5B substitutions [32-37] were studied in 266 Venezuelan HCV isolates from chronically infected, drug-naïve patients (n = 127 and n = 228 for core and NS5B respectively), and compared to isolates from other countries (n = 355 and n = 646 for core and NS5B respectively).

Core mutations R70Q and L/C91M, associated with IR [12], increased HCC risk [13-17], variable responses to double (IFN/RBV) [18-24] and triple (teleprevir plus INF/RBV) therapy [25,26], and liver steatosis [27,28], were mainly found in G1b isolates. In Venezuelan isolates, 42% carried one and 53% both substitutions (data not shown). These mutations were significantly more frequent in American isolates compared to Asian ones (Table 1). G1a, G2b and G3a presented a Cysteine in position 91 while G2c and G2j a Leucine. In general, both in core and NS5B region, G2j polymorphisms were essentially similar to those found in G2c. Steatosis motives in core domain 3 (amino acids 182 and 186 [29,30]) were exclusive to G3a.

**Table 1:** Frequency of amino acid substitution 70Q and 91 M of core protein in HCV Venezuelan isolates compared to worldwide isolates

**Genotype**	**Geographic region**	**R70Q substitution**	**L/C91M substitution**
**1a**	**V**	1/27 (3.7%)	0/27 (0%)
	**U**	1/27 (3.7%)	0/27 (0%)
	**J**	0/5 (0%)	0/5 (0%)
	**Ur**	0/3 (0%)	0/3 (0%)
**1b**	**V**	30/38 (79%)	26/38 (68%)
	**U**	20/34 (65%)	28/34 (82%)
	**Am**	**50/72 (69%)**	**54/72 (75%)**
	**J**	32/80 (40%)	25/80 (31%)
	**Ch**	7/70 (10%)	43/70 (61%)
	**As**	**39/150 (26%)**	**68/150 (45%)**
		**p < 0.001**	**p < 0.001**
**2a**	**J**	0/66 (0%)	0/66 (0%)
**2b**	**V**	0/3 (0%)	0/3 (0%)
	**J**	0/32 (0%)	0/32 (0%)
**2c**	**V**	0/7 (0%)	0/7 (0%)
	**RW**	0/20 (0%)	0/20 (0%)
**2j**	**V**	0/42 (0%)	0/42 (0%)
**3a**	**V**	1/10 (10%)	0/10 (0%)
	**RW**	2/18 (11%)	0/18 (0%)

V: Venezuela; U: USA; Ur: Uruguay; Am: American countries (USA and Venezuela). As: Asian countries like Ch: China and J: Japan (including prevalence data of Furui et al. [39], for which sequences are not available); RW: Rest of the world. Numbers in bold refer to mutations found at higher frequency compared to other genotypes or locations. P: Statistical significance of prevalence of mutations in isolates from the Americas compared to Asia.

In NS5B region, D310N, D244N, T329I and S326G substitutions have been described to be induced by RBV treatment [33]. D310N was detected mainly in G3a (100%) and G1a (13%) Venezuelan samples (Table 2). Interestingly, the presence of this mutation in G1a isolates was significantly higher than that found in Brazilian isolates (p = 0.03). D244N was only found in G3a (78% in Venezuela). G2 harbored a Serine in this position. T329I and S326G could only be analyzed in G2 and G3 isolates, since the length of G1 sequences did not allow the analysis of this region. T329I was only found in G2b isolates (100%), T329A was frequent in G2c and G2j (93% and 90% respectively), while T329V was found in G3a (100%). S326G was no found in the HCV isolates analyzed.

**Table 2:** Frequency of amino acid substitution in NS5B protein related to resistance to INF/RBV and new inhibitors in HCV Venezuelan isolates compared to worldwide isolates

	**Genotypes**								
	**1a**			**1b**			**3a**		
**Substitution (related to)**	**V**	**Br**	**U**	**V**	**LA**	**ASIA**	**V**	**LA**	**U**
**Q309R (INF/RBV)**	36/82 (44%)	**67/101 (66%) p = 0.004**	28/70 (40%)	7/61 (12%)	7/104 (6.7%)	19/335 (5.7%)	9/9 (100%)	20/22 (91%)	21/21 (100%)
**D310N (RBV)**	**11/82 (13%) p = 0.03**	4/101 (4%)	3/70 (4%)	1/61 (1.6%)	1/104 (1%)	3/335 (0.9%)	**9/9 (100%)**	**20/22 (91%)**	**20/21 (95%)**
**S282T (NI)**	0/82 (0%)	0/101 (0%)	0/70 (0%)	0/61 (0%)	0/104 (0%)	0/335 (0%)	0/9 (0%)	0/22 (0%)	0/21 (0%)
**C316N (NNI)**	0/82 (0%)	0/101 (0%)	0/70 (0%)	11/61 (18%)	18/104 (18%)	**307/335 (91.6%) p < 0.001**	0/9 (0%)	0/22 (0%)	0/21 (0%)

V: Venezuela; Br: Brazil; LA: Other Latin-American countries (Brazil, Chile, Colombia and/or Argentina); U: USA; Asia: China, Japan, Hong Kong and Taiwan. Numbers in bold refer to mutations found at higher frequency compared to other genotypes or locations. P: Statistical significance of prevalence of mutations in isolates from Venezuela compared to Brazil (for Q309R and D310N) and from Venezuela and Latin America compared to Asia (C316N).

Q309R and A333E NS5B mutations, frequents in patients with SVR and end-of-treatment response (ETR) after INF/RB therapy [32], were also analyzed. Q309R mutation was present in all genotypes (100% G3a, 44% G1a and 12% G1b from Venezuela), except G2 which harbored a Valine in this position (100% G2a, G2b, G2c and 91% G2j). This mutation was more frequently found in Brazilian G1a isolates compared to Venezuelan and USA isolates (Table 2). A333E mutation, only analyzed in Venezuelan G2 and G3 isolates, was present in most G2 samples (100% G2a, G2b, G2c and 98% G2j from Venezuela). G3a samples harbored Lysine or Arginine substitution at this position.

In addition, in the NS5B region, S282T and C316Y/N substitutions, associated to resistance to new NS5B inhibitors (2^′^-C-methyl modified ribonucleosides and HCV-796, respectively) [34-37], were also analyzed. S282T mutation (Table 2) and C316Y mutation were not found in any of the HCV isolates analyzed. However, C316N polymorphism was only detected in 18% of Venezuelan G1b isolates, in a similar prevalence to that found in other Latin American countries, but with a significantly lower frequency than those found in Japan (Table 2).

**Figure 1:**
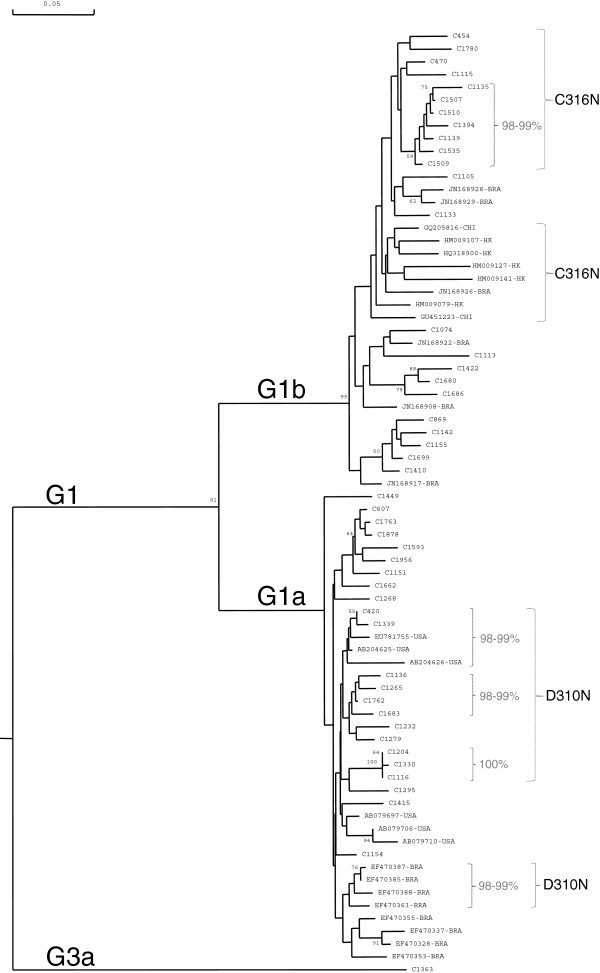
**Phylogenetic analysis of the NS5B partial genomic region (256 nt, 8302–8556) of HCV G1 strains.** Genetic distance was estimated by Kimura 2 parameters and phylogenetic tree was constructed with the Neighbor joining method. Bootstrap values over 50% are shown in the tree. Isolates are designated by their GenBank accession number, followed by their country of origin, except for Venezuelan ones, which are numbered and preceded by a C. G1a sequences harboring D310N substitution were included, and a similar number of sequences not harboring these mutations, from Venezuela (n = 23), Brazil (BRA, n = 8) and USA (n = 6). The same criteria of selection was applied to G1b sequences, respect to C316N substitution, from Venezuela (n = 23), Brazil (n = 6) and Asia (n = 7 from HK: Hong Kong, JAP: Japan and CHI: China). Sequences grouped in clades containing D310N or C316N substitutions are shown in brackets. Percent identities over 98% are shown for these clades.

Phylogenetic analysis was performed in order to evaluate the genetic relatedness of G1 isolates harboring D310N and C316N substitutions in the NS5B region (Figure 1). Interestingly, most of the HCV isolates harboring these variants, and particularly G1a isolates with D310N substitution, were associated in clades, displaying more than 98% of identity. However, G1a Venezuelan isolates harboring these mutations did not group with the Brazilian ones. Some of the clades were composed of isolates from one country, while one clade included isolates from Venezuela and USA (Figure 1).

Others mutations that also confers resistance to others NNIs, have been identified downstream the NS5B region [38]. These mutations could not be analyzed in this study, due to the length of the sequence analyzed.

## Discussion

A total of 12 amino acid positions were analyzed in core [12-30] and/or NS5B region [32-37] of Venezuelan HCV isolates and compared to worldwide isolates. Substitutions in these sites have been related either to IFN/RBV treatment or differential susceptibility to others drugs, and/or to a worse prognosis of the disease [12-30,32-37]. These substitutions were found in 9/12 positions analyzed, with genotypical, geographical and/or regional differences in the prevalence of them.

R70Q and L/C91M core substitutions were found more frequently in Venezuelan and USA G1b isolates, compared to Asian ones. These amino acid substitutions in core region of different HCV genotypes are associated with increased HCC risk [13-17], variable responses to IFN/RBV therapy [18-24], liver steatosis [27-30] and IR [12]. Treatment with IFN/RBV has been proposed to induce these amino acid mutations [23]. Another possible explanation is that these mutations might be selected during the natural course of infection, in response to the selective pressure of endogenous IFN [39]. The relatively high frequency of these mutations in patients not treated with IFN/RBV might be associated to the transmission of HCV isolates from treated patients. In addition, this relative high frequency in Latin America warrants further follow up studies in patients harboring one or two of these mutations. Steatosis motives in core domain 3 were exclusive to G3a, as previously reported [29].

Some mutations have been described in the NS5B region and appeared to be generated during IFN/RBV treatment, probably because of the mutagenic effect of RBV [33]. D310N was detected in all G3a isolates. Interestingly, the frequency of this mutation was higher in Venezuelan G1a compared to Brazilian and USA isolates. Phylogenetic analysis of G1a isolates showed that the isolates carrying D310N were grouped in clades with high genetic relatedness, in agreement with the hypothesis of networks of transmission between non-treated patients infected with HCV isolates from a treated patient.

In addition, some of these mutations have been found more frequently in viruses infecting patients with sustained virological response (SVR) or during end-of-treatment response (ETR), in which the virus has been persistently or at least temporarily eliminated [32]. From these, the Q309R and A333E mutations were analyzed in this study. In Venezuelan isolates Q309R was present in all genotypes except G2, very frequent in G3 and rare in G1b. In G1a the frequency of this mutation was similar in Venezuelan and USA isolates, but significantly lower than those found in Brazil. Instead, A333E was frequent in G2 and rare in G3, while it could not be analyzed in G1. These results are in agreement with the frequency found in other countries [40]. The presence of these amino acid substitutions might be related to the relative susceptibility to IFN/RBV of G2 and G3 compared to G1.

S282T mutation in the NS5B polymerase is known to confer resistance to the inhibitor 2^′^-C-methyl modified ribonucleosides [34] with a decrease viral fitness [35]. This mutation was not present in the isolates analyzed in this study.

For NNIs, resistance mutations may also occur as natural variants more frequently in patients infected with a specific subtype. For example, the C316Y mutation in NS5B is associated most frequently with resistance to HCV-796 [36]. Furthermore, C316N also reduces sensitivity to HCV796 *in vitro* and this polymorphism is observed in many patients infected with HCV subtype 1b [41-43]. While C316Y mutation was not present in the isolates analyzed, C316N variant was detected in 18% of Venezuelan G1b isolates, with a significantly lower prevalence than in Asian isolates, where this mutation was found at high frequency (Table 2). Again, phylogenetic analysis showed that Venezuelan G1b isolates carrying the C316N were generally grouped in clades, displaying high genetic relatedness.

Two major clades have been described inside G1a isolates worldwide [44]. Brazilian isolates belong mainly to one of these clades [45], and even form a cluster inside this clade [46]. Venezuelan HCV isolates do not group together with Brazilian isolates (Figure 1). These results suggest that important regional variations might be found in HCV isolates circulating in South America. An example of this is the predominant circulation of G2j among the G2 isolates circulating in Venezuela, and not found in other neighboring countries [7].

## Conclusions

Genotypical, geographical and regional differences were found in the prevalence of substitutions in HCV core and NS5B proteins. The substitutions found in the Venezuelan G2j type were similar to that found in G2a and G2c isolates. Our results suggest a high prevalence in Venezuela of the R70Q and L/C91M mutations of core protein for G1b and D310N substitution of NS5B protein for the G3a. As expected, C316N polymorphism, associated to resistance to NNIs was only found in G1b, and mutation S282T to NIs was absent. However, the presence of mutations associated to a worse prognosis of the disease in HCV-infected patients warrants further studies to analyze their impact in the clinical outcome of this disease in Venezuela.

## Methods

### Blood samples

Serum samples were collected from 1997 to 2010, from HCV-infected untreated patients after written informed consent, and stored at −30°C until use. This study was approved by the Bioethical Committee of Instituto Venezolano de Investigaciones Cientificas (IVIC). A total of 127 Venezuelan samples (27 infected with HCV-1a, 38 HCV-1b, 3 HCV-2b, 7 HCV-2c, 42 HCV-2j and 10 with HCV-3a) were processed for core amplification (57.5% male) and 228 (82 infected with HCV-1a, 61 HCV-1b, 1 with HCV-2a, 13 HCV-2b, 9 HCV-2c, 53 HCV-2j and 9 with HCV-3a) for NS5B amplification (58.3% male).

### PCR and sequencing

HCV RNA was extracted from human plasma sample using a QIAamp® Viral Mini Kit (QIAGEN, Hilden Germany) and reverse-transcribed to complementary DNA using MMLV Reverse Transcriptase (Invitrogen life technologies, USA), according to the manufacturers protocol. HCV genotype was determined by direct sequencing and phylogenetic analysis of a polymerase chain reaction-amplified product from the *5'* non-coding region [6], core and/or NS5B [7]. The NS5B amplicons were generated previously [7]. The core amplified PCR products were purified by using the QIAquick PCR Purification Kit (Qiagen) and then subjected to direct nucleotide sequencing. In all cases, both sense and antisense inner primers were used for sequencing and all sequences were performed by Macrogen Service Center, Seoul, Korea. A total of 127 core sequences were analyzed: 49 obtained previously [7] and 79 from this study.

### Determination and analysis of core and NS5B amino acid sequence

Amino acid sequences were deduced and aligned using MEGA 4.0.2. The NS5B sequences [GenBank accession numbers HM777048-HM777357] and core sequences [GenBank accession numbers HM777360 -HM777430 and JQ924868-JQ924946] of Venezuelan HCV-infected naïve patients were analyzed. The sequences of each genotype from other countries were obtained from GenBank and HCV database (http:VHC.lanl.gov/content/VHC-db/index).

### Phylogenetic analysis

Sequence alignment performed by the global alignment algorithm, using DNAman 5.2.2 (Lynnon Bio Soft, Canada). Phylogenetic analysis was performed by the Neighbor Joining method (1000 bootstrap replicas, with genetic distances estimated with Kimura 2 parameters correction).

### Statistical analysis

Statistical differences were evaluated by the Chi-Squares test with Yates correction, or Fisher Exact test, according to a computerized Epi Info program, version 3.5.3 (Centers for Disease Control and Prevention, Atlanta, GA).

## Abbreviations

ETR: End-of-treatment response; G: Genotype; HCC: Hepatocellular carcinoma; HCV: Hepatitis C virus; IFN: Pegylated Interferon alpha; IR: Insulin resistance; NIs: Nucleoside inhibitors; NNIs: Non- nucleoside inhibitors; RBV: Ribavirin; SVR: Sustained virological response.

## Competing interests

The authors manifest non competing interests.

## Authors’ contributions

RCJ, YFS, MZS, CLL, HRR, and FHP carried out the molecular genetic studies. RCJ, YFS and FHP participated in the sequence alignment and phylogenetic analysis. RCJ and FHP drafted the manuscript. All authors read and approved the final manuscript.
